# Educational attainment in offspring bereaved by sudden parental death from external causes: a national cohort study from birth and throughout adulthood

**DOI:** 10.1007/s00127-020-01846-4

**Published:** 2020-02-13

**Authors:** Lisa Victoria Burrell, Lars Mehlum, Ping Qin

**Affiliations:** National Centre for Suicide Research and Prevention, University of Oslo, Norway, Sognsvannsveien 21, 0372 Oslo, Norway

**Keywords:** Parental bereavement, Educational attainment, Cohort study, Population registers

## Abstract

**Purpose:**

Previous research has linked loss of a parent during childhood to reduced educational aspirations, school performance, and educational attainment later in life. The potential effect of maternal and paternal bereavement on attainment at all educational levels is, however, unknown. The present study aimed to investigate the potential influence of parental death by external causes on completion of compulsory education, high school, vocational education, and University or College education.

**Methods:**

The study was based on data from three national longitudinal registers in Norway. The study population comprised 373,104 individuals born between January 1st 1970 and December 31st 1994. Information concerning deceased parents’ cause and date of death and offspring’s education and sociodemographic data were retrieved. Data were analysed with Cox regression.

**Results:**

Children who had experienced parental death by external causes had a significantly reduced hazard ratio (HR) of completing all educational levels compared to children who did not have such experiences. The largest effects were evident for completion of high school (HR 0.68, 95% CI 0.65–0.71) and University or College education (HR 0.75, 95% CI 0.70–0.80). No differences were evident for different causes of death, genders of deceased or ages at bereavement, and generally no significant interactions between gender of the bereaved offspring and predictor variables were evident for completion of all educational levels.

**Conclusion:**

Parental death by external causes has vast and long-lasting impacts on offspring’s educational attainment at all levels. Health care interventions aimed at supporting bereaved children and adolescents should focus on challenges related to educational progress.

## Introduction

The sudden death of a parent from an accident, suicide or homicide constitutes one of the most traumatic and significant life events a child or adolescent can experience [1]. Related to such a traumatic loss, offspring have an increased risk of severe psychosocial problems, including psychiatric disorders, marital dissolution, violent crime and suicide [2–4]. Parental loss has also been found to negatively affect offspring’s school performance and educational attainment: Reduced likelihood of enrolment and graduation as well as lower mean grades [5–7] is accompanied by reduced expectations and aspirations for future education and career [8] in bereaved children compared to children who have not experienced the loss of a parent. Furthermore, poor school performance is again associated with psychosocial and somatic problems [9–11] which further impairs school performance, effectively creating a negative spiral or developmental cascade [12]. Higher education, on the other hand, is associated with higher life satisfaction and happiness [13].

Several previous studies investigating educational attainment in parentally bereaved children and adolescents have been restricted to paternal bereavement [5, 7, 14, 15], or have used data from the US [8, 16–18] where all higher education is based on tuition fees paid by students and/or their families. This complicates generalization to most European countries where students and families to a lesser extent have to pay for education. A recent register-based cohort study by Berg and colleagues [6] investigated both maternal and paternal bereavement in the Swedish population and found that parental death was associated with a lower probability of school graduation in offspring aged 16 years. What the subsequent educational trajectory will be after bereaved offspring have finished the final compulsory school year at age 16 is still unclear.

The present register-based cohort study aims to expand current knowledge by investigating the influence of parental death by external causes during childhood and adolescence on completion of compulsory education, high school, vocational education, and University or College education. We, furthermore, aim to study the effects of specific causes of parental death, genders of the deceased parent and ages at bereavement on educational attainment into adulthood. External causes of death refer to deaths where the cause is external to the body, such as accidents, suicides and homicides, and is a classification of mortality in the ICD coding system. Given the aims of the study, we did not include parental death due to natural causes.

## Methods

### Data sources

We retrieved individual data from three Norwegian longitudinal registers and merged them by means of the personal identification number. The first register is the Central Population Register, which has been computerized since 1964 and contains demographic data and a personal identification number for all individuals residing in Norway. The register also contains a link to parents, which was utilized in order to identify the mother and father of the cohort members. The second register is the Cause of Death Register, which has been computerized since 1969 and contains the cause and date of all deaths in Norway coded according to ICD-8 (International Classification of Deceases, Eight Revision) from 1969 to 1985, ICD-9 from 1986 to 1995 and ICD-10 from 1996 to 2012 [19]. Last, the third register is Statistics Norway’s Events Database (the so-called FD-Trygd database) which contains demographic and socioeconomic data, such as information concerning education and ethnicity. Data on education in this register is based on the National Education Database [20], and encompasses education statistics at an individual level dating back to 1970 [21].

The study was approved by the Regional Ethical Committee for Medical and Health Research (REK sør-øst) and owners of the relevant registers. Informed consent from participants was deemed unnecessary and impossible by the ethical committee because this was a population-based study with de-identified register data.

### Study design and population

The present study is a retrospective cohort study which aims to investigate whether exposure is associated with outcome incidence through continuous observation over time [22]. The use of a cohort study and cox regression analyses enabled the investigation of educational attainment across the subject’s age span in order to analyse both the likelihood of attainment and its age dispersion. The study population consisted of a 25% random sample of all Norwegian residents born between January 1st 1970 and December 31st 1994 who had a link to both their father and mother in the Central Population register. The cohort comprised 373,104 individuals.

### Variables of interest

#### Educational attainment

The present study investigated four outcomes related to educational attainment at all the different educational levels in the Norwegian school system: completion of compulsory education after age 15, and high school, vocational education and University or College education after age 18. The educational information was retrieved from Statistics Norway’s Events Database [23].

First, completion of *compulsory education after age 15* (lower secondary education) was investigated. The compulsory education in Norway consists of 7 years at primary school and 3 years at secondary school, and all children are automatically enrolled in this education. The education is normally completed the year children turn 16 years.

We furthermore investigated completion of *high school after age 18* (post-secondary non-tertiary education). This education can either focus on preparation for further academic studies, or on becoming a craftsman within a particular craft, industry or service. The former education often takes 3 years, while the latter often takes 4 years or more. Completion of compulsory education is required for attending high school, and every youth in Norway who have completed compulsory education have the right to attend high school.

Completion of *vocational education after age 18* (short-cycle tertiary education) was also investigated. Vocational education is a further education within a particular craft, and it builds on the craft studied at high school for the individuals who chose this educational direction. The education can take between 6 months and 2 years, and completion of high school is required for attending vocational education.

Last, we investigated completion of *University or College education after age 18*. This education includes one-year programs, bachelor’s degree programs, and master’s degree programs, which all require completion of a high school education. If an individual had completed several University or College degrees, the date of the first degree was included in the analyses. Doctoral degrees were not included in the analyses because PhD students in Norway are employed and receive a standard salary and social welfare benefits on the same level as all other Norwegian employees.

#### Parental bereavement

The explanatory variable of interest in the study is exposure to parental death by external causes during childhood, referred to as parental DBEC (codes E800-E999 in ICD-8 and ICD-9 and V01-Y89 in ICD-10). When examining completion of compulsory education, the effect of parental DBEC before age 15 was investigated, while the effect of parental DBEC before age 18 was investigated when examining completion of high school, vocational education, and University or College education. Data concerning potential maternal and/or paternal DBEC were retrieved from the Cause of Death Register, and subjects were classified into two categories of *bereavement status* as (a) no exposure to parental DBEC or (b) exposure to parental DBEC. Specific *causes of parental death* were further classified as (a) suicide, (b) transport accident (including land, water and air transport methods), and (c) other external causes (such as other accidents, homicide and injury with unknown intent). *Gender of deceased parent* was classified as (a) father, (b) mother, and (c) both parents. Subjects were classified according to their *age at bereavement* into (a) ≤ 4 years, (b) 5–9 years, (c) 10–14 years, and (d) 15–18 years. If both parents died, cause of death and age at bereavement were classified according to the parent who died first since this marks the beginning of children’s bereavement-related exposure.

#### Covariates

Several covariates were controlled for in the multivariate analyses. Ethnicity was classified as (a) born in Norway with two Norwegian born parents, (b) immigrant, (c) born in Norway with immigrant parents or one parent born abroad, and (d) born abroad with one or two Norwegian born parents. Additionally, analyses were adjusted for cohort members’ year of birth due to the change in the level of educational attainment over time, and gender due to gender differences in educational attainment.

### Statistical analyses

The study cohort was followed from birth to date of educational attainment at the different educational levels, or date of death, emigration from Norway or at most December 31st 2012, whichever came first. Differences in hazard ratios (HRs) with concomitant 95% confidence intervals were estimated using Cox regression analyses. The potential effects of bereavement variables on completion of the different educational levels were estimated in the crude models, while the adjusted models included all covariates, i.e. ethnicity, year of birth and gender. Since completion of compulsory education is required for attending high school, high school completion was also adjusted for completion of compulsory education in the multivariate analyses. Similarly, since graduating from high school is required for attending vocational education and University or College education, completion of these educational levels was adjusted for completion of high school in the multivariate analyses. Sensitivity analyses restricting the sample to individuals who have completed the required educational level, rather than controlling for previous completion, were also performed. In these analyses, the start of follow-up was set to the date of graduating from the required education. Interactions between variables of study with gender were assessed with the log likelihood ratio test based on results from the multivariate analyses. All analyses were conducted in Stata, version 15 [24].

## Results

In the current cohort of 373,104 individuals, 51.4% (191,840) were males, and 48.6% (181,264) were females. Before age 18, 3844 people were censored due to death and 9837 people were censored due to emigration. Overall, 3692 individuals (1918 males and 1774 females) had experienced parental death by external causes before age 18. Distribution of the study variable categories in the cohort by completion of the different educational levels is presented in Table 1. For compulsory education, 95.1% of people who had not experienced parental death by external causes completed the education, while 95.8% of people who had been exposed to parental death by external causes completed this education. The corresponding percentages were 67.2% and 56.3% for completing high school, 3.9% and 3.1% for completing vocational education, and 33.4% and 24.9% for completing University or College education. The mean age for completion of compulsory education was 16.0 years for exposed and non-exposed offspring, while the mean age for completion of high school was 19.9 years for offspring who had not experienced parental DBEC and 20.4 years for offspring who had experienced such loss. The corresponding mean ages were 23.4 and 24.7 for completion of vocational education, and 23.5 and 24.1 for completion of University or College education.

**Table 1:** Distribution (%) of the study variable categories in the cohort, by completion of the different educational levels

Variable	Total (*N* = 373,104)		Completed compulsory education (*N* = 354,902)^a^	Completed high school (*N* = 250,389)^b^	Completed vocational education (*N* = 14,349)^b^	Completed University or college education (*N* = 124,274)^b^
	Bereaved age 15	Bereaved age 18				
**Bereavement status**						
No exposure to parental DBEC	370,123	369,412	352,045 (95.1)	248,309 (67.2)	14,235 (3.9)	123,355 (33.4)
Exposure to parental DBEC	2981	3692	2857 (95.8)	2080 (56.3)	114 (3.1)	919 (24.9)
**Cause of death**						
No exposure to parental DBEC	370,123	369,412	352,045 (95.1)	248,309 (67.2)	14,235 (3.9)	123,355 (33.4)
Suicide	1160	1452	1127 (97.2)	846 (58.3)	41 (2.8)	346 (23.8)
Transport accident	965	1126	912 (94.5)	649 (57.6)	37 (3.3)	313 (27.8)
Other external causes	856	1114	818 (95.6)	585 (52.5)	36 (3.2)	260 (23.3)
**Gender of deceased**						
No exposure to parental DBEC	370,123	369,412	352,045 (95.1)	248,309 (67.2)	14,235 (3.9)	123,355 (33.4)
Father	2422	2949	2347 (96.9)	1690 (57.3)	97 (3.3)	747 (25.3)
Mother	498	672	469 (94.2)	363 (54.0)	17 (2.5)	157 (23.4)
Both parents	61	71	41 (67.2)	27 (38.0)	0 (0)	15 (21.1)
**Age at bereavement**						
No exposure to parental DBEC	370,123	369,412	352,045 (95.1)	248,309 (67.2)	14,235 (3.9)	123,355 (33.4)
< 4 years	904	904	851 (94.1)	495 (54.8)	21 (2.3)	222 (24.6)
5–9 years	974	974	928 (95.3)	533 (54.7)	38 (3.9)	232 (23.8)
10–14 years	1103	1103	1078 (97.7)	649 (58.8)	28 (2.5)	282 (25.6)
15–18 years		711		403 (56.7)	27 (3.8)	183 (25.7)

^a^The percentages of completion was based on bereavement before age 15

^b^The percentages of completion was based on bereavement before age 18

**Fig. 1:**
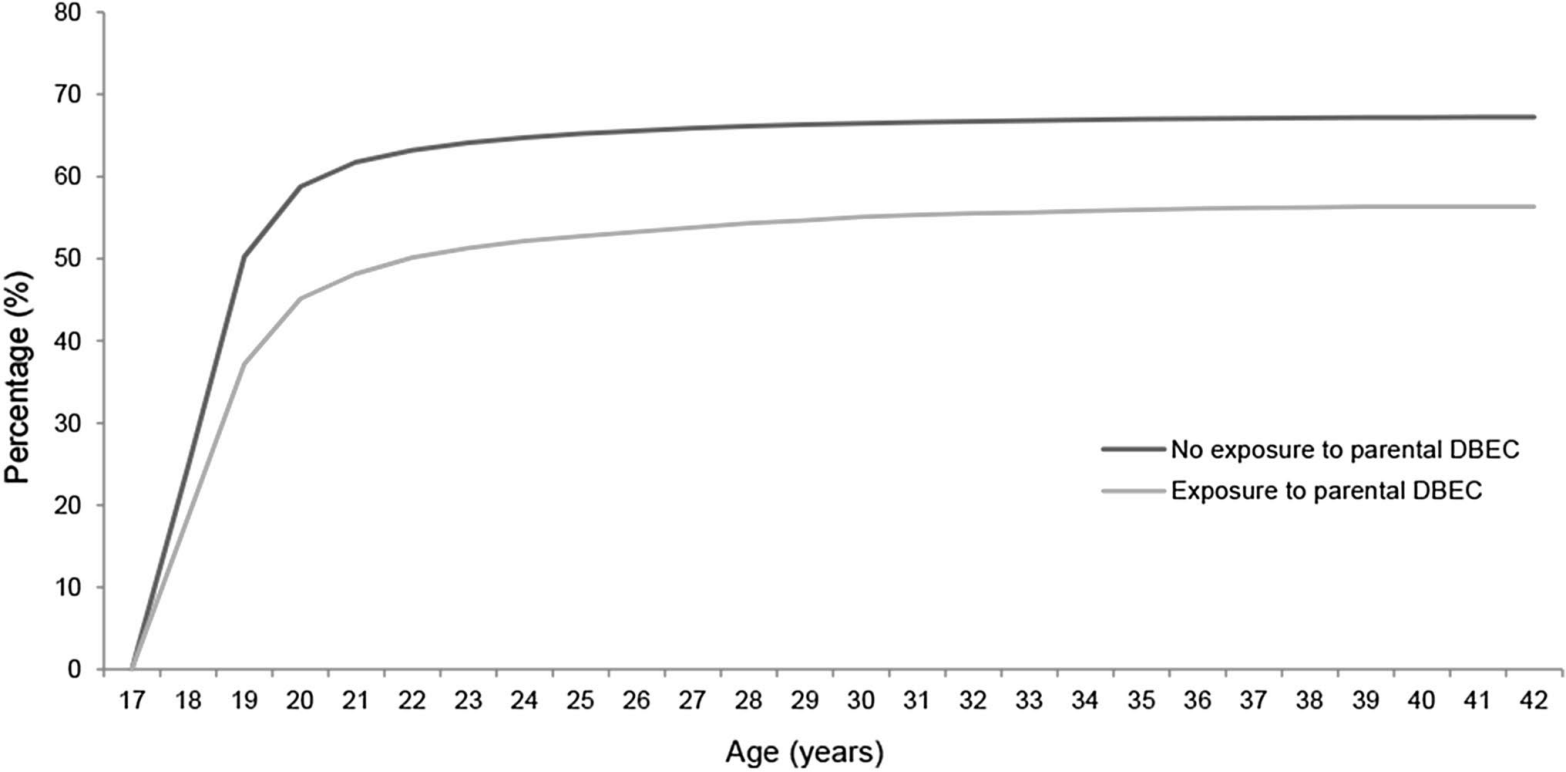
Percentage of completion of high school by offspring’s age for the different bereavement groups

**Fig. 2:**
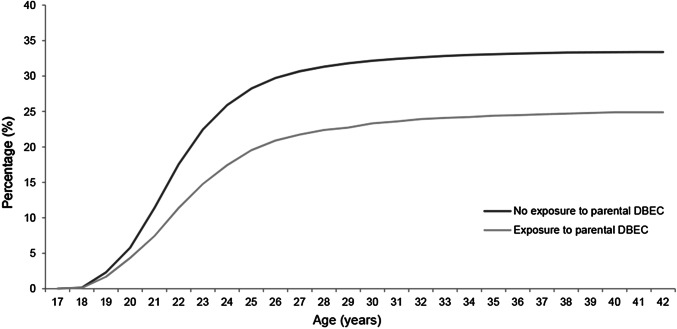
Percentage of completion of University or College education by offspring’s age for the different bereavement groups

Table 2 presents the crude and adjusted HRs with 95% confidence intervals for completion of the different educational levels associated with the variables under study. In the multivariate analyses adjusted for ethnicity, gender, year of birth and the prerequisite educational level, individuals who had experienced parental death by external causes had significantly lower HRs of completing all educational levels compared to individuals who had not experienced parental DBEC: Compulsory education (HR 0.94, 95% CI 0.91–0.98), high school (HR 0.68, 95% CI 0.65–0.71), vocational education (HR 0.78, 95% CI 0.65–0.94), and University or College education (HR 0.75, 95% CI 0.70–0.80). The percentage of completion of high school and University or College education by offspring’s age for the different bereavement groups is visualized in Figs. 1 and 2, respectively, again depicting the difference in completion between exposed and non-exposed individuals. As is evident from the figures, the difference between exposed and non-exposed offspring is sustained into adulthood.

**Table 2:** Crude and adjusted HRs with 95% confidence intervals for completion of the different educational levels associated with the variables under study

Variable	Crude HR				Adjusted HR			
	Compulsory education	High school	Vocational education	University or college education	Compulsory education^a^	High school^b^	Vocational education^c^	University or college education^c^
**Bereavement status**								
Exposure to parental DBEC	0.98 (0.95–1.02)	0.69 (0.66–0.72)‡	0.77 (0.64–0.93)†	0.66 (0.62–0.71)‡	0.94 (0.91–0.98)†	0.68 (0.65–0.71)‡	0.78 (0.65–0.94)†	0.75 (0.70–0.80)‡
**Cause of death**								
Suicide	1.00 (0.94–1.05)	0.72 (0.67–0.77)‡	0.72 (0.53–0.98)*	0.64 (0.58–0.71)‡	0.95 (0.90–1.01)	0.71 (0.66–0.76)‡	0.72 (0.53–0.98)*	0.70 (0.63–0.78)‡
Transport accident	0.96 (0.90–1.02)	0.70 (0.65–0.76)‡	0.78 (0.56–1.08)	0.71 (0.64–0.79)‡	0.92 (0.86–0.98)*	0.69 (0.64–0.75)‡	0.79 (0.57–1.09)	0.81 (0.72–0.90)‡
Other external causes	1.00 (0.93–1.07)	0.64 (0.59–0.70)‡	0.84 (0.61–1.17)	0.64 (0.57–0.73)‡	0.96 (0.90–1.03)	0.63 (0.58–0.69)‡	0.86 (0.62–1.19)	0.75 (0.66–0.85)‡
**Gender of deceased**								
Father	0.97 (0.94–1.01)	0.69 (0.66–0.73)‡	0.81 (0.66–0.99)*	0.66 (0.61–0.71)‡	0.93 (0.90–0.97)‡	0.68 (0.65–0.72)‡	0.82 (0.67–1.00)*	0.75 (0.69–0.80)‡
Mother	1.05 (0.96–1.15)	0.69 (0.62–0.76)‡	0.67 (0.42–1.08)	0.67 (0.58–0.79)‡	1.02 (0.93–1.11)	0.68 (0.61–0.75)‡	0.67 (0.42–1.08)	0.76 (0.65–0.89)‡
Both parents	0.81 (0.60–1.10)	0.62 (0.42–0.90)*	-	0.75 (0.45–1.24)	0.78 (0.57–1.05)	0.62 (0.43–0.91)*	–	0.89 (0.54–1.47)
**Age at bereavement**								
≤ 4 years	1.00 (0.93–1.07)	0.68 (0.62–0.74)‡	0.59 (0.38–0.90)*	0.67 (0.59–0.77)‡	0.94 (0.88–1.01)	0.67 (0.61–0.73)‡	0.60 (0.39–0.91)*	0.78 (0.68–0.89)‡
5–9 years	0.92 (0.86–0.98)†	0.65 (0.60–0.71)‡	0.97 (0.71–1.34)	0.61 (0.54–0.70)‡	0.89 (0.83–0.95)‡	0.65 (0.59–0.70)‡	0.98 (0.71–1.35)	0.72 (0.63–0.82)‡
10–14 years	1.04 (0.98–1.10)	0.73 (0.68–0.79)‡	0.63 (0.44–0.92)*	0.68 (0.61–0.77)‡	1.00 (0.94–1.06)	0.72 (0.66–0.77)‡	0.64 (0.44–0.93)*	0.74 (0.66–0.83)‡
15–18 years		0.71 (0.64–0.78)‡	0.95 (0.65–1.38)	0.70 (0.60–0.81)‡		0.69 (0.63–0.76)‡	0.97 (0.66–1.41)	0.78 (0.67–0.90)‡

No exposure to parental DBEC as reference

^*^p < .05, †*p* < .01, ‡*p* < .001

^a^The HRs derived from these models were adjusted for ethnicity, year of birth, and gender

^b^The HRs derived from these models were adjusted for ethnicity, year of birth, gender and completion of compulsory education

^c^The HRs derived from these models were adjusted for ethnicity, year of birth, gender, and completion of high school

Sensitivity analyses restricting the sample to individuals who have completed compulsory education showed that individuals exposed to parental DBEC still had significantly lower HRs of completing high school (HR 0.69, 95% CI 0.66–0.72). Likewise, exposed individuals still had significantly lower HRs of completing a University or College education when the sample was restricted to high school graduates (HR 0.79, 95% CI 0.74–0.85). For vocational education, however, there was no longer a difference between the exposed and unexposed groups when restricting the sample to individuals who had completed high school (HR 0.87, 95% CI 0.69–1.08).

### Specific causes of parental death

Parental bereavement from transport accidents was associated with a significantly reduced HR of completing compulsory education in the multivariate analysis, while parental suicide and other external causes of death did not lead to a significantly decreased HR of completion. For completion of high school, all external causes of death were associated with a significantly reduced HR of completing. Parental suicide was the only specific cause of death associated with a significantly reduced HR of completing vocational education, while all causes of death were associated with significantly reduced HRs of completing University or College education. The category of other external causes of death consisted largely of accidents (84.6%), such as poisonings (23.9%) and falls (15.4%), and homicide (11.1%). In general, no large differences depending on cause of death were evident for any educational level.

### Gender of deceased parent

No large differences depending on the gender of the deceased parent were evident for any educational level. Paternal bereavement was associated with a significantly reduced HR of completing all levels of education, while maternal bereavement was associated with significantly reduced HRs of completing high school and University or College education. Bereavement of both parents was associated with significantly reduced HRs of completing high school.

### Age at bereavement

For compulsory education, bereavement between the ages of 5 and 10 years was associated with a significantly reduced HR of completion, while all ages of bereavement were associated with significantly reduced HRs of completing high school. Bereavement before age 5 years and between 10 and 15 years was associated with reduced HRs of completing vocational education, and all ages of bereavement were associated with significantly reduced HRs of completing University or College education. In general, no large differences were evident depending on offspring’s age at bereavement for any educational level.

### Gender of bereaved offspring

No significant interactions between gender of the bereaved offspring and bereavement status were evident for all educational levels, indicating that parental DBEC reduced the HR of completing all educational levels equally for daughters and sons (data not shown). Log likelihood ratio tests for interactions between gender of the offspring and cause of death, gender of deceased and age at bereavement for all educational levels only resulted in one significant interaction between gender and cause of death for completion of compulsory education. Evidently, parental suicide and transport accidents reduced the HRs of completing compulsory education slightly more in sons than in daughters (data not shown).

## Discussion

To our knowledge, this is the first study to investigate the potential effect of maternal and paternal bereavement by external causes on completion of all educational levels. The present study shows that children and adolescents who have experienced parental death by external causes have a significantly lower probability of completing all educational levels, from compulsory education to University or College education, compared to individuals who have not experienced parental DBEC. The largest effects were evident for completion of high school and University or College education. Furthermore, no large differences were evident depending on cause of death, gender of deceased parent, age at bereavement, and gender of bereaved offspring.

### Association between bereavement status and educational attainment

The present study adds to previous literature stating that individuals who have experienced parental death by external causes have an increased risk of suffering from a range of unfortunate psychosocial challenges such as psychiatric disorders, marital dissolution, suicide, and criminal activity [2–4, 25]. In addition to a reduced likelihood of completing compulsory education, similar to what has been previously reported by Berg and colleagues [6], bereaved children have a significantly reduced probability of attaining all educational levels. A previous Norwegian register study investigating paternal death by all causes, however, only reported a significant difference between bereaved and non-bereaved children for completion of compulsory education and high school, but not completion of University or College education [7]. As opposed to the present study, this study did not include maternal bereavement and investigated all causes of death.

Potential reasons for the reduced probability of educational attainment in bereaved offspring may be found in a combination of pre- and post-bereavement factors. Relevant pre-bereavement factors preceding the loss probably include lower household income and parental education [26], parental psychiatric illness [8, 27], and an associated turbulent family environment. Post-bereavement factors probably consist of offspring’s psychiatric disorders caused or aggravated by the loss [28, 29], lower family cohesion and social support [8], and challenges specifically related to education, such as impaired concentration [30], lower sense of mastery and self-esteem [31], and lower school attendance [32]. This study is unfortunately unable to investigate the influence of these pre- and post-bereavement factors on offspring’s educational attainment due to data limitations.

### Cause of death

Previous studies have investigated potential differences in school performance between children bereaved by parental death from external causes and parental death from natural causes [5, 6], but no study to date has differentiated between various external causes of death. Clearly, the higher suicide risk in offspring who have been bereaved by parental suicide compared to parental death by other external causes [2, 25, 33] does not transfer to a difference in educational attainment given that the present study failed to identify differences across all educational levels when comparing different external causes of death. External causes of death have several similarities in that they happen suddenly, leaving the bereaved with no or very little chance to prepare themselves or say goodbye, and are accompanied by shock, drama, and a potentially traumatic impact. These types of death may induce a sense of loss of control in the bereaved, are often associated with strong sensory impressions or fantasies, and may lead to rumination and counterfactual thoughts about the death. Additionally, many problems that typically trouble suicide bereaved families have in fact also been found to influence families where a parent has died from other causes, such as low household income and education, high family discord, and psychiatric impairment in the adult caregiver [8, 26, 34]. Likewise, even though suicide is often preceded by mental illness, accidental deaths, especially poisonings and falls, are also often associated with pre-existent mental disorders [27, 35].

### Gender of deceased parent and bereaved offspring

The present study did not find any significant difference in educational attainment at any level by gender of the deceased parent, and minimal differences by gender of the bereaved offspring. These findings are in accordance with previous studies reporting similar educational sequela following maternal and paternal bereavement [6], and loss in daughters and sons [5, 6, 18]. The lack of gender differences is also in accordance with our recent study from Norway reporting no differences in bereaved offspring’s suicide risk depending on gender of the deceased parent and gender of the bereaved offspring [25].

The similar impact of parental bereavement regardless of gender of the deceased parent and bereaved offspring may reflect a high level of gender equality in Norway today. The role of primary caregiver, displays of affection and attachment, and other factors that have traditionally differed between mothers and fathers may now be more individually based and hence differ between different families [36, 37]. As a consequence, both parents may function as primary attachment figures, or the remaining parent may be willing and able to take on the role as primary attachment figure following the loss of the co-parent. Moreover, economic and occupational gender equality [38] may enable the remaining parent to more effectively uphold household resources in the absence of the co-parent. Gender equality may also explain the lack of significant differences in the mourning experiences of bereaved daughters and sons due to more equal ability to express their feelings and receive social support.

### Age at bereavement

Evidently, losing a parent throughout childhood and adolescence is detrimental to educational attainment. The lack of large differences depending on children’s age at bereavement corresponds well with previous studies reporting similar effects for different ages at bereavement for school performance and educational attainment [6, 7]. In a similar vein, a recent large-scale register study in Norway reported an especially increased suicide risk in offspring bereaved by parental death by external causes during childhood and adolescence [25], again indicating the importance of these age periods as developmentally critical periods [39]. Even though we do not see large effects depending on children’s age at bereavement, different developmental processes may be responsible for determining offspring’s educational attainment at varying ages of bereavement. Post-bereavement factors may account for educational challenges following bereavement in early childhood, such as family discord, offspring’s psychiatric disorders and developmental difficulties associated with growing up in a one-parent household [28, 29, 34, 40, 41]. On the other hand, pre-bereavement factors may be more influential when bereavement occurs in later adolescence, including parental psychiatric disorders and poor socioeconomic status [8, 26]. Challenges directly related to education, such as impaired concentration, lower sense of mastery and self-esteem, and lower school attendance and follow-up at home are probably influential following bereavement at all developmental periods [30–32].

### Strengths and limitations

The major strengths of the present study include the long follow-up time of offspring from birth and throughout adulthood and the investigation of all educational levels from compulsory education until University or College education. Moreover, the utilization of national longitudinal registers enables a large sample size, effectively increasing statistical power when investigating a rare event such as parental death by external causes. Data in Norwegian registers cover the entire population and are collected systematically and uniformly. The data quality has been found to be high [42, 43] and the registers are continuously monitored and corrected [44, 45]. Register studies do not suffer from problems related to sampling and attrition, and eliminate biases such as recall bias. Last, cohort studies such as the present study are able to infer causal relationships to a much larger extent than case–control or cross-sectional studies [46].

Results from the present study are naturally subject to limitations, chief among them being the lack of information concerning family socioeconomic status and parental psychiatric disorders, enabling identification of the potential effects of these underlying factors. Since a requirement for inclusion in the study was a registered link to both mother and father in the Central Population Register, our sample probably consisted of fewer individuals with an immigration background compared to the frequency in the population as a whole. Last, to ensure that bereavement occurred before the outcome under investigation, the cut-off age at bereavement was set to 15 years for compulsory education and 18 years for later educations. This will to a degree underestimate the number of people who have experienced parental DBEC, and some offspring exposed before finishing the education in question will be classified as un-exposed. This limitation will, however, lead to a type II error and hence reduce the chance of significant findings.

### Conclusions and implications

In conclusion, individuals who have experienced parental death by external causes during childhood and adolescence have a significantly lower probability of completing all educational levels compared to individuals who have not experienced parental DBEC, regardless of cause of death, gender of deceased parent, age at bereavement, and gender of bereaved offspring. Parental death by external causes evidently has vast and long-lasting impacts on offspring’s educational attainment, and the educational level may be hard to improve in adulthood.

The findings from the present study suggest that schools at all educational levels should offer more educational support services to students who have experienced bereavement in order to prevent educational decline. Educational difficulties add to the psychosocial problems already found to be associated with parental bereavement, and the somatic, psychological, social, and developmental challenges experienced by bereaved offspring may manifest as a negative spiral or developmental cascade. As a result, health care interventions aimed at supporting bereaved children and adolescents should not only focus on psychological challenges, but also challenges related to familial and interpersonal problems and educational progress, especially due to the detrimental impact of low socioeconomic status on health risk behaviours, drug abuse, mental health problems, somatic diseases, and even suicide [9–11, 47]. The comprehensive and interlinked nature of the extensive psychosocial sequela following parental death from external causes stress the importance of understanding and treating bereavement-related challenges in association.

## References

[1] Bowlby J (1980). Attachment and loss.

[2] Wilcox HC, Kuramoto SJ, Lichtenstein P, Langstrom N, Brent DA, Runeson B (2010). Psychiatric morbidity, violent crime, and suicide among children and adolescents exposed to parental death. J Am Acad Child Adolesc Psychiatr.

[3] Gravseth HM, Mehlum L, Bjerkedal T, Kristensen P (2010). Suicide in young Norwegians in a life course perspective: Population-based cohort study. J Epidemiol Community Health.

[4] Høeg BL, Johansen C, Christensen J, Frederiksen K, Dalton SO, Dyregrov A, Bøge P, Dencker A, Bidstrup PE (2018). Early parental loss and intimate relationships in adulthood: a nationwide study. Dev Psychol.

[5] Prix I, Erola J (2017). Does death really make us equal? Educational attainment and resource compensation after paternal death in Finland. Soc Sci Res.

[6] Berg L, Rostila M, Saarela J, Hjern A (2014). Parental death during childhood and subsequent school performance. Pediatrics.

[7] Steele F, Sigle-Rushton W, Kravdal O (2009). Consequences of family disruption on children's educational outcomes in Norway. Demography.

[8] Brent D, Melhem NM, Masten AS, Porta G, Payne MW (2012). Longitudinal effects of parental bereavement on adolescent developmental competence. J Clin Child Adolesc Psychol.

[9] Gauffin K, Vinnerljung B, Fridell M, Hesse M, Hjern A (2013). Childhood socio-economic status, school failure and drug abuse: a Swedish national cohort study. Addiction.

[10] Jablonska B, Lindberg L, Lindblad F, Rasmussen F, Ostberg V, Hjern A (2009). School performance and hospital admissions due to self-inflicted injury: a Swedish national cohort study. Int J Epidemiol.

[11] Link BG, Phelan J (1995). Social conditions as fundamental causes of disease. J Health Soc Behav.

[12] Masten AS, Cicchetti D (2010). Developmental cascades. Dev Psychopathol.

[13] Higher Education Funding Council for England (2017) The wellbeing of graduates: assessing the contribution of higher education to graduates’ wellbeing in the UK. http://dera.ioe.ac.uk/id/eprint/30632. Accessed 10 Nov 2019

[14] Biblarz TJ, Gottainer G (2000). Family structure and children's success: A comparison of widowed and divorced single-mother families. J Marriage Fam.

[15] Fronstin P, Greenberg DH, Robins PK (2001). Parental disruption and the labour market performance of children when they reach adulthood. J Popul Econ.

[16] Amato PR, Anthony CJ (2014). Estimating the effects of parental divorce and death with fixed effects models. J Marriage Fam.

[17] Williams LD, Aber JL (2016). Testing for plausibly causal links between parental bereavement and child socio-emotional and academic outcomes: a propensity-score matching model. J Abnorm Child Psychol.

[18] Feigelman W, Rosen Z, Joiner T, Silva C, Mueller AS (2017). Examining longer-term effects of parental death in adolescents and young adults: evidence from the national longitudinal survey of adolescent to adult health. Death Stud.

[19] Statistics Norway (2012) Causes of death - About the statistics. https://www.ssb.no/en/helse/statistikker/dodsarsak/aar/2013-11-01?fane=om#content. Accessed 8 Jan 2016

[20] Akselsen A, Lien S, Sivertstøl Ø (2005) FD-Trygd Variabelliste [Statistics Norway's Events Database Variabel list]. https://www.ssb.no/a/publikasjoner/pdf/notat_200717/notat_200717.pdf. Accessed 26 May 2019

[21] Statistics Norway (2017) National education database. https://www.ssb.no/en/omssb/tjenester-og-verktoy/data-til-forskning/utdanning/om-nasjonal-utdanningsdatabase Accessed 22 May 2019

[22] Veierød MB, Lydersen S, Laake P, Veierød MB, Lydersen S, Laake P (2012). Design and analysis. Medical statistics in clinical and epidemiological research.

[23] Statistics Norway (2018) Database for standard classifications. https://stabas.ssb.no/MainFrames.asp?Language=en. Accessed 15 Jan 2018

[24] StataCorp (2017) Stata Statistical Software: Release 15 StataCorp LLC College Station, TX

[25] Burrell LV, Mehlum L, Qin P (2017). Sudden parental death from external causes and risk of suicide in the bereaved offspring: A national study. J Psychiatr Res.

[26] Fauth B, Thompson M, Penny A (2009). Associations between childhood bereavement and children’s background, experiences and outcomes.

[27] Melhem NM, Walker M, Moritz G, Brent DA (2008). Antecedents and sequelae of sudden parental death in offspring and surviving caregivers. Arch Pediatr Adolesc Med.

[28] Kaplow JB, Saunders J, Angold A, Costello EJ (2010). Psychiatric symptoms in bereaved versus nonbereaved youth and young adults: a longitudinal epidemiological study. J Am Acad Child Adolesc Psychiatr.

[29] Shear K, Clayton PJ (2008). Bereavement-related depression. Psychiatr Ann.

[30] Dyregrov K, Dyregrov A (2007). Sosial nettverksstøtte ved brå død [Social network support following sudden death].

[31] Marks NF, Jun H, Song J (2007). Death of parents and adult psychological and physical well-being—a prospective US national study. J Fam Issues.

[32] Cas AG, Frankenberg E, Suriastini W, Thomas D (2014). The impact of parental death on child well-being: evidence from the Indian ocean tsunami. Demography.

[33] Guldin MB, Li J, Pedersen HS, Obel C, Agerbo E, Gissler M, Cnattingius S, Olsen J, Vestergaard M (2015). Incidence of suicide among persons who had a parent who died during their childhood: a population-based cohort study. JAMA Psychiatry.

[34] Maier EH, Lachman ME (2000). Consequences of early parental loss and separation for health and well-being in midlife. Int J Behav Dev.

[35] Crump C, Sundquist K, Winkleby MA, Sundquist J (2013). Mental disorders and risk of accidental death. Br J Psychiatry.

[36] Statistics Norway (2012) Tidene skifter: Tidsbruk 1971–2010 [Times are changing: use of time 1971–2010]. https://www.ssb.no/a/publikasjoner/pdf/sa125/sa125.pdf. Accessed 8 Nov 2019

[37] Hamre K (2017) Fedrekvoten—mer populær enn noen gang [Paternal quota—more popular than ever]. Samfunnsspeilet 1, Statistics Norway. https://www.ssb.no/befolkning/artikler-og-publikasjoner/fedrekvoten-mer-populaer-enn-noen-gang--298200. Accessed 8 Nov 2019

[38] World Economic Forum (2016) The global gender gap report, Geneva, Switzerland. http://www3.weforum.org/docs/WEF_GGGR_2020.pdf. Accessed 8 Nov 2019

[39] Kuh D, Ben-Shlomo Y, Lynch J, Hallqvist J, Power C (2003). Life course epidemiology. J Epidemiol Community Health.

[40] Ogata K, Ishikawa T, Michiue T, Nishi Y, Maeda H (2011). Posttraumatic symptoms in japanese bereaved family members with special regard to suicide and homicide cases. Death Stud.

[41] Shear K, Mulhare E (2008). Complicated Grief Psychiatr Ann.

[42] Pedersen AG, Ellingsen CL (2015). Datakvaliteten i dødsårsaksregisteret [the data quality in the cause of death register]. Tidsskrift Norsk Legeforening.

[43] Tøllefsen IM, Helweg-Larsen K, Thiblin I, Hem E, Kastrup MC, Nyberg U, Rogde S, Zahl PH, Østevold G, Ekeberg Ø (2015). Are suicide deaths under-reported? Nationwide re-evaluations of 1800 deaths in Scandinavia. BMJ Open.

[44] Erlangsen A, Qin P, Mittendorfer-Rutz E (2018). Studies of suicidal behavior using national registers An adventure without boundaries?. Crisis.

[45] Norwegian Institute of Public Health (2016) Cause of death statistics. https://www.fhi.no/en/hn/health-registries/cause-of-death-registry/cause-of-death-registry-/. Accessed 21 May 2019

[46] Ahrens W, Krickeberg K, Pigeot I, Ahrens W, Pigeot I (2007). An introduction to epidemiology. Handbook of epidemiology.

[47] Qin P, Agerbo E, Mortensen PB (2003). Suicide risk in relation to socioeconomic, demographic, psychiatric, and familial factors: a national register-based study of all suicides in Denmark, 1981–1997. Am J Psychiat.

